# Workplace bullying and its relationship with spiritual climate: a latent profile analysis

**DOI:** 10.3389/fpsyg.2025.1516345

**Published:** 2025-05-05

**Authors:** Rong Huang, Xu Ran, Chenxi Tang, Yanlin Wei

**Affiliations:** ^1^The Second Clinical Medical College of North Sichuan Medical College, Nanchong, China; ^2^Beijing Anzhen Nanchong Hospital, Capital Medical University & Nanchong Central Hospital, Nanchong, Sichuan, China

**Keywords:** workplace bullying, standardized training for nurses, potential profile analysis, spiritual climate, influencing factors

## Abstract

**Objective:**

To explore the group characteristics and heterogeneity of workplace bullying among standardized training of nurses, to find out the potential categories of workplace bullying, and to explore its relationship with spiritual climate, to more comprehensively understand the occupational environment and mental health of standardized training of nurses, and to provide theoretical basis for managers to develop effective intervention measures.

**Methods:**

The multi-center cross-sectional study method and 648 nurses in eight Grade A hospitals in Sichuan province were selected using the Chinese negative behavior questionnaire and spiritual climate questionnaire. Latent profile analysis was used to identify the heterogeneity of workplace bullying among standardized training of nurses and to use multiple linear regression to explore the relationship between workplace bullying and spiritual climate.

**Results:**

According to the results of potential profile analysis, standardized nurses can be divided into three potential categories: “severe workplace bullying group,” “no workplace bullying group” and “bad workplace situation group.” According to the general data of one-way ANOVA, age, average weekly working hours, love of nursing work, harmonious relationship with colleagues, and whether formal work before training were the main factors affecting training nurses’ feeling of bullying in the workplace (*p* < 0.05, *p* < 0.01). Multiple linear regression analysis showed a negative association between spiritual climate and workplace bullying, with lower workplace bullying scores and higher spiritual climate scores.

**Conclusion:**

The proportion of organizational dimensions and bad workplace situations is too high. Nursing managers and educators should pay attention to and analyze the causes of bullying of standardized training nurses, and take effective preventive measures to reduce the occurrence of workplace bullying. Bullying in the workplace affects the spiritual climate, so nursing managers should consider formulating relevant management plans from the organizational level to reduce the occurrence of bullying in the workplace and improve the spiritual climate of the department.

## 1 Introduction

Standardized training (hereinafter referred to as regular training) refers to the systematic clinical training after completing basic studies, which is an important way to cultivate clinical nursing talent echelon (He et al., 2023; Ding et al., 2020). Since the implementation of the (General Office of the National Health Commission, 2016) regulation and training measures of the National Health Commission in 2016, more and more nursing students have chosen standardized training after graduation from school. Unstable working environment, unfamiliar interpersonal relationship and lack of professional ability, lack of work experience, imperfect psychological defense mechanism, leading to nurses who are in training of standardized be more prone to workplace bullying between nurses (Ji et al., 2019). Chinese mainland nurse About 39.59–53.33% suffered bullied (Li et al., 2023), and the incidence of bullying in standardized training of nurses is about 47.9% (Ji et al., 2019). Workplace bullying (workplace bullying) refers to the malicious, long-term, repetitive negative behavior in the workplace, which is one of the most stressful phenomena in the workplace (Zhu et al., 2023; Chen and Zheng, 2020). Fox and Spector (2005) proposed a stressor-emotion theory model based on the frustration-attack hypothesis and occupational stress theory, which believed that individual attitudes and behaviors towards work are influenced by work environment factors and individual factors. Individual perception of the environment leads to the emergence of related emotions, which then induce behavioral tendencies. Workplace bullying is one of the most common social stressors, affecting nurses’ cognitive evaluation of the work environment, leading to negative emotional reactions such as frustration (Matheson and Bobay, 2007). At the same time, when nurses think that they are unfair in the working environment, they will produce unpleasant emotions such as anger and shame, and the process of regulating negative emotions will reduce the ability of self-control, resulting in antiproductive behavior, which adversely affects the outcome of patient safety and nursing quality (Guglielmi et al., 2018; Akter, 2019). Bullying in the workplace not only affects the physical and mental health of the bullied nurses, which is not conducive to the long-term development of nursing personnel training, but also affects the nursing quality of patients and damages the healthy operation of the medical care environment (Zhu et al., 2023). Potential profile analysis is a statistical method to screen the classification based on strict objective adaptation index (Tein et al., 2013). Workplace bullying is a frequent, dynamic, multivariate social phenomenon with types and consequences of complexity and diversity (Acquadro et al., 2024). In this study, we used potential profile analysis and combined with various evaluation indicators to explore workplace bullying in regulation nurses, which was more accurate, objective and scientific to find out the influencing factors affecting workplace bullying in regulation nurses. Spiritual climate refers to the spiritual and cultural atmosphere of working environment, which encourages employees to express their inner world, promotes cooperation and communication between individuals and team members, so as to build a trust relationship and share inner experience in the context of work. This atmosphere is an invisible positive force that shapes the workplace communication and interaction model (Zhang et al., 2019; You et al., 2020). Duan et al. (2023) and other scholars believe that the impact of hospital and department environment on bullying in nurses’ workplace cannot be ignored. The relationship between the department’s spiritual climate and workplace bullying is still unknown. This study through the potential profile analysis method to explore the potential categories and characteristics of the workplace, more carefully divide the bullying of the workplace, and explore the relationship with the spiritual climate, which will help us to get a more comprehensive understanding of the occupational environment and mental health, to improve the working environment of nurses, reduce the risk of bullying, improve their work enthusiasm and satisfaction, to develop effective interventions.

## 2 Objects and methods

### 2.1 Study subjects

From August 2024 to September 2024, nurses who are participating in the standardized training from 8 tertiary hospitals in Sichuan province were selected as the survey objects. Inclusion criteria: ① To obtain the nurse practice qualification. ② Is participating in the standardized training of nurses in Sichuan province. ③ Volunteer to participate in this project survey. Exclusion criteria: out of duty during the survey (including maternity leave, sick leave or regular nurses during the extended training phase).

### 2.2 Sample size calculation

The general data of this study involved 19 variables, the negative behavior questionnaire contained 22 items, and the spiritual climate contained 4 items, for a total of 45 variables. In cross-sectional studies, the sample size is generally required as more than 5–10 times of the number of independent variables, and considering 20% invalid responses, the sample size is calculated to be 563 cases. In this study, 648 nurses, 75 boys and 573 girls in Sichuan province.

### 2.3 Methods

#### 2.3.1 Survey contents

① Demographic data: including gender, age, height, education background, marital status, harmonious relationship with colleagues, monthly income, weekly working hours, etc. ② Bullying in the workplace: Use the negative behavior questionnaire (the Negative Acts Questionnaire Revised, NAQ-R), which was compiled and revised by Einarsen et al. (2003), and is mainly used to measure bullying in the workplace. The Chinese version of the questionnaire was translated and revised by Xun et al. (2012), including 22 items in 3 dimensions: personal-related bullying (9 items), work-related bullying (4 items) and organizational injustice (4 items). Using the Likert5 scoring method, 1,2,3,4,5 for “never,” “occasionally,” “monthly,” “weekly” and “daily,” respectively, with higher scores indicating a higher level of perceived bullying in the workplace. The questionnaire Cronbach’s *α* coefficient ranged from 0.954 to 0.959, and the content validity index was 0.956. ③ Chinese version of spiritual climate brief scale (Chinese Version of the Spritual Climate Scale, C-SCS): compiled by Janel D. Sexton and J. Bryan Sexton (Wu et al., 2019). The Chinese into the Chinese version of spiritual climate brief scale. The scale includes 4 items, with 1 to 5 points from “very disagree” to “very agree” and a total score from 4 to 20. The higher the score, the higher the level of spiritual climate perception. The Cronbach’s α coefficient of this scale was 0.83.

#### 2.3.2 Investigation method

The electronic questionnaire survey is conducted by using the questionnaire star, all the contents of the questionnaire is input into the questionnaire star software, and the electronic questionnaire is made. Each item is set as a required answer, and the unified instruction instructions are added. Before the investigation, after obtaining the consent of the nursing department of the hospital, the regular nurses were organized to establish a wechat group, and explained the investigation purpose, research content and questionnaire filling requirements to the respondents in detail, and then the respondents filled in the survey by themselves. In order to ensure the authenticity and reliability of the data and the representativeness and universality of the sample, the regular nurses in the grade A hospitals in different regions were selected as the survey objects. This study will be conducted strictly according to confidentiality principles, in an anonymous manner and with an informed consent option to ensure that participants’ personal information and privacy are not violated. In order to avoid repeated filling, the same mobile phone number or computer IP address can only be filled in once. Statistical data shall be checked by two persons and analyzed in strict accordance with statistical methods and principles to ensure the authenticity of the data. The researchers collected the survey data through the questionnaire star platform, and eliminated the questionnaires that did not meet the standards.

#### 2.3.3 Statistical methods

In this study, SPSS 26.0 software and Mplus 8.7 software were used for statistical analysis, Results were considered as significant when indicated as two-sided *p* < 0.05. The general data of the nurses was described using the frequency and percentage; Nurses’ workplace bullying, mental climate use mean, and standard deviation for descriptive analysis; and the three dimensions of NAQ-R: personal-related negative behavior, organizational injustice and work-related negative behavior as a measure of workplace bullying. Latent profile analysis was used to fit the potential categories of bullying in the nurses’ workplace. One-way analysis of variance and *χ* 2 test was used to compare the distribution differences in the dimensions and general data of nurses; linear regression analysis was used to compare the differences in spiritual climate of nurses.

## 3 Results

A total of 648 questionnaires were distributed in this study, and all of the questionnaire results were valid.

### 3.1 General data

The study object is 648 standardized training nurses in Sichuan province, among them, 75 (11.6%) were male, 573 (88.4%) were women; height distribution: ≤155 cm,66 persons (10.18%), 155 ~ 160 cm, 259 persons (39.97%), 160 ~ 165 cm, 195 persons (30.09%), > 165 cm, 128 persons (19.75%); age distribution: ≤20 years old, 6 persons (0.92%), 21 ~ 25 years old, 614 persons (94.75%), 26 ~ 30 years old, 28 persons (4.32%) (see Table 1 for more details).

**Table 1:** General data the study object (*n* = 648).

Project	Number	Proportion
Gender		
Male	75	11.6%
Women	573	88.4%
Stature		
<155 cm	66	10.18%
155 ~ 160 cm	259	39.97%
161 ~ 165 cm	195	30.09%
>165 cm	128	19.75%
Age		
<20 years old	6	0.92%
20 ~ 25 years old	614	94.75%
26 ~ 30 years old	28	4.32%
Marital status		
Unmarried	625	96.45%
Married	23	3.54%
Educational background		
Junior college	356	54.93%
Undergraduates	292	45.06%
The professional title		
Nurses	633	97.68%
Primary nurse	15	2.31%
The standardized training grade		
the first grade	347	53.55%
the second grade	301	46.45%
Employment form		
the unit commissioned the training	138	21.3%
the society commissioned the training	510	78.7%
The number of nurses in the department		
<15 persons	66	10.18%
15 ~ 25 persons	217	33.49%
26 ~ 35 persons	106	16.36%
> 35 persons	259	39.97%
The city		
Nanchong city	176	27.16%
Suining city	8	1.23%
Deyang city	148	22.84%
Zigong city	85	13.12%
Yibin city	223	34.41%
Ya’an city	8	1.23%
The average weekly working hours		
<40 h	173	26.7%
40 ~ 50 h	415	64.04%
> 50 h	60	9.26%
Attitudes towards the nursing work		
Passionate	216	33.33%
Neutral	399	61.57%
Loath	33	5.09%
Relationship with colleagues		
Get along well with their colleagues	437	67.44%
Neutral	205	31.63%
Did’t get along well with their colleagues	6	0.92%
Average monthly income		
<3,000	359	55.4%
3,000 ~ 5,000	254	39.2%
> 5,000	35	5.4%
Work experience before the training		
Has been officially hired	78	12.04%
Have not been formally employed	570	87.96%
Self personality type		
Outgoing character	181	27.93%
Introverted character	172	26.54%
Neutral easygoing type	295	45.52%

### 3.2 Workplace bullying score

Workplace bullying score perceived by nurses in Sichuan province measured by NAQ-R workplace bullying score of 22–109, with the total score (28.1 ± 8.99); the scores of each dimension are: personal related negative behavior (10.87 ± 3.37), work-related negative behavior (11.84 ± 4.19) and organizational injustice score (5.37 ± 1.96), detailed in Table 2.

**Table 2:** Score of all dimensions of workplace bullying in Sichuan province (*n* = 648).

Project	Total mean score (x ± s)	The item is evenly divided (x̅ ± s)
Personal related bullying	10.87 ± 3.37	1.20 ± 0.37
Work-related bullying	11.84 ± 4.19	1.32 ± 0.47
Unfair organization	5.37 ± 1.96	1.34 ± 0.49
total points (TP); aggregate score	28.10 ± 8.99	1.27 ± 0.41

### 3.3 Potential profile analysis of bullying in the workplace of nurses who are participating in the standardized training

#### 3.3.1 Identify the best model

Five latent category models were constructed, with AIC, BIC and aBIC values decreasing as categories increased, while all Entropy values were > 0.8. Among the five models, the Entropy value of model 5 was the highest, and the AIC, BIC and aBIC values were the smallest, but the LMRT value was not statistically significant. Considering the LMRT value of model 4 and the significant likelihood of model 3, and the Entropy value of model 3 is higher than the other three models, from the perspective of clinical application, the best model of bullying in the workplace is the 3 category model (see Table 3 for more details).

**Table 3:** Potential profile analysis of the different workplace bullying categories.

Number of classes	Log-likelihood	AIC	BIC	ABIC	Entropy	LMRT	BLRT	Class probability
1	−12223.798	24539.596	24745.324	24599.276	-	-	-	-
2	−9283.452	18706.903	19019.967	18797.720	0.992	0.640	0.000	0.8662 0.1338
3	−7865.157	15918.313	16338.714	16040.267	0.997	0.0253	0.000	0.0108 0.8364 0.1528
4	−7221.158	14678.315	15206.052	14831.406	0.927	0.7123	0.000	0.0046 0.0938 0.8212 0.0804
5	−6594.984	13473.968	14109.042	13658.196	0.998	0.7232	0.000	0.0046 0.0930 0.8220 0.0696 0.0108

AIC = Akaike Information Criterion Akaike information criterion; BIC = Bayesian Information Criterion Bayesian information criterion; aBIC = Adjusted Bayesian Information Criterion adjusted Bayesian information criterion; LMR = Lo–Mendell–Rubin; BLRT = Bootstrap Likelihood ratio test bootstrapping likelihood ratio test.

#### 3.3.2 Classification results and naming of each category of the best model

**Figure 1:**
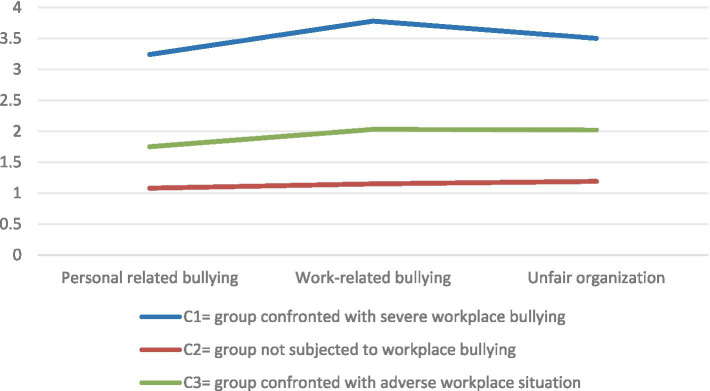
Analysis of the potential profile of nurse workplace bullying.

Seven regulatory nurses in the C1 category, 1%, minal proportion, the average score of workplace bullying in this category was 3.24, 3.78 points, 3.50 points, all were above 2 points, show that C1 class training nurses experience severe workplace bullying at work, therefore, the category was named as the “Severe Workplace Bullying Group”; there are 542 training nurses in the C2 category, for 84%, the largest proportion, In addition, the average score of workplace bullying among the nurses in this category was 1.08, 1.15, and 1.19, respectively, all were below 2 points, therefore, the category was named as the “no workplace bullying group”; 99 training nurses in the C3 category, accounting for 15%, the average score of the three dimensions of workplace bullying was 1.75, 2.03, and 2.02, respectively, among them, work-related negative behavior and organizational injustice dimension scored more than 2 points, therefore, the category was named “Encounter adverse workplace ConSituation Group,” Workplace bullying was also considered to have occurred (see Figure 1).

#### 3.3.3 Univariate analysis of the potential categories of workplace bullying

A one-way ANOVA was used to compare the scores between the three potential section scores in each dimension and the total score (*p* < 0.001). Post-hoc comparison results showed that the scores of nurses in the (C2) were significantly lower than” group confronted with severe workplace bullying “(C1) and (C3); nurses in the (C3) were significantly higher than” group not subjected to workplace bullying “(C2), and significantly lower than nurses in the” severe workplace bullying “(C1) (see Table 4 for details).

**Table 4:** Comparison of score differences in different categories and dimensions of workplace bullying (x̅ ± s).

Dimension	Workplace bullying potential category			*P*	Posterior comparisons
	C1	C2	C3		
Personal related bullying	29.14 ± 7.62	9.74 ± 1.18	15.80 ± 2.94	<0.001	C1 > C3 > C2
Work-related bullying	34.00 ± 7.16	10.39 ± 1.60	18.25 ± 3.26	<0.001	C1 > C3 > C2
Unfair organization	14.00 ± 3.60	4.77 ± 1.07	8.09 ± 1.90	<0.001	C1 > C3 > C2
Total score	77.14 ± 16.26	24.90 ± 3.13	42.15 ± 5.98	<0.001	C1 > C3 > C2

C1 = group confronted with severe workplace bullying, C2 = group not subjected to workplace bullying, C3 = group confronted with adverse workplace situation.

#### 3.3.4 Differences in general information on the potential categories of bullying regulatory training nurses in the workplace

Using a one-way ANOVA, To compare the differences in the general data between the three potential categories of training nurses, The results showed that different potential categories of workplace bullying trained nurses at age (*F* = 9.998, *p* < 0.001), average weekly working hours (*F* = 21.623, *p* < 0.001), whether they love nursing work (*F* = 16.759, *p* < 0.001), whether the harmonious relationship with colleagues (*F* = 73.955, *p* < 0.001), whether formal worked before training (*F* = 4.880, *p* = 0.008) was statistically significant (see Table 5).

**Table 5:** Differences in general data for workplace bullying [*n* (%)].

Variable	Workplace bullying potential category			F-number	P
	C1	C2	C3		
Age					
≤20 years old	0	6 (1.1)	0	9.998	<0.001
21–25 years old	6 (85.7)	521 (96.1)	87 (87.9)		
26–30 years old	1 (14.3)	15 (2.8)	12 (12.1)		
Average weekly working hours					
≤40 h	0	159 (29.3)	7 (7.1)	21.623	<0.001
41–50 h	4 (57.1)	341 (62.9)	70 (70.7)		
>50 h	3 (42.9)	42 (7.7)	22 (22.2)		
Whether you love nursing work					
Love	0	201 (37.1)	15 (15.2)	16.759	<0.001
Commonly	4 (57.1)	320 (59.0)	75 (75.8)		
Do not love	3 (42.9)	21 (3.9)	9 (9.1)		
Have a good relationship with your colleagues					
Harmonious	0	408 (75.3)	29 (29.3)	73.955	<0.001
Commonly	3 (42.9)	133 (24.5)	69 (69.7)		
Not harmonious	4 (57.1)	1 (0.2)	1 (1.0)		
Whether you have worked formally before the training					
Yes	3 (42.9)	58 (10.7)	17 (17.2)	4.880	0.008
No	4 (57.1)	484 (89.3)	82 (82.8)		

C1 = group confronted with severe workplace bullying, C2 = group not subjected to workplace bullying, C3 = group confronted with adverse workplace situation.

### 3.4 Descriptive analysis of the spiritual climate

The total score of bullying in the workplace (15.24 ± 3.13). The scores of each item are shown in Table 6.

**Table 6:** Spiritual climate score situation (x̅ ± s).

Variable	Score
My department encourages and supports my opinions and ideas, and my colleagues can listen to and accept them	3.76 ± 0.89
Doctors and nurses respect my opinions and ideas	3.75 ± 0.84
When I share my ideas with my colleagues, I feel a sense of belonging and identity	3.83 ± 0.85
In my department, everyone can express their own ideas. We respect each other and understand each other	3.91 ± 0.86
Total score	15.24 ± 3.13

### 3.5 Univariate analysis of workplace bullying and spiritual climate for nurses who are participating in the standardized training

The results of univariate analysis showed that there were significant differences in the items and total scores of workplace bullying (*p* < 0.001). Different workplace bullying potential categories of spiritual climate items and total score after comparison shows: “not suffer workplace bullying group” (C2) rules of spiritual climate of the item score and total score is significantly higher than “severe workplace bullying group” (C1) and “encounter workplace bad situation group” (C3) rules of nurse (see Table 7).

**Table 7:** Univariate analysis of potential categories of workplace bullying and spiritual climate (x̅ ± s).

Variable	Agree with the situation	Workplace bullying potential category			*P*	Posterior comparisons
		C1	C2	C3		
My department encourages and supports my opinions and ideas, and my colleagues can listen to and accept them	Disagree	5 (71.5%)	24 (4.4%)	10 (10.1%)	<0.001	C2 > C3 > C1
	Neutral	2 (28.5%)	118 (21.8%)	64 (64.6%)		
	Agree	0 (0%)	400 (73.8%)	25 (25.3%)		
Doctors and nurses respect my opinions and ideas	Disagree	6 (85.7%)	16 (2.9%)	6 (6%)	<0.001	C2 > C3 > C1
	Neutral	1 (14.3%)	137 (25.3%)	67 (67.7%)		
	Agree	0 (0%)	389 (71.89%)	26 (26.3%)		
When I share my ideas with my colleagues, I feel a sense of belonging and identity	Disagree	4 (57.1%)	16 (2.9%)	10 (4.6%)	<0.001	C2 > C3 > C1
	Neutral	3 (42.9%)	106 (19.6%)	53 (25%)		
	Agree	0 (0%)	420 (77.5%)	36 (70.4%)		
In my department, everyone can express their own ideas. We respect each other and understand each other	Disagree	5 (71.4%)	13 (2.4%)	14 (14.1%)	<0.001	C2 > C3 > C1
	Neutral	2 (28.6%)	74 (13.7%)	46 (46.5%)		
	Agree	0 (0%)	455 (83.9%)	39 (39.4%)		

C1 = group confronted with severe workplace bullying, C2 = group not subjected to workplace bullying, C3 = group confronted with adverse workplace situation.

### 3.6 Linear regression analysis of workplace bullying potential category and spiritual climate

With spiritual climate score as dependent variables, in the control of age, the average weekly work hours, whether love nursing work, relationship with colleagues, whether have worked formally before the training after confounding factors, multiple linear regression analysis was performed with the workplace bullying potential category as the independent variable (set dummy variables, to suffer serious workplace bullying as reference group). The results showed that compared with the nurses in (C1), the spiritual climate scores of (C2) (B = 8.335, *p* < 0.001) and (C3) (B = 5.521, *p* < 0.001) were still significantly higher than the (C1), 17.1% of the explained total variation, as shown in Table 8.

**Table 8:** Linear regression analysis of workplace bullying potential categories and spiritual climate.

Project	Coefficients	*t*	*p*
(constant)	7.429	6.905	0.000
Group confronted with severe workplace bullying	-	-	-
Group not subjected to workplace bullying	8.335	7.698	0.000
Group confronted with adverse workplace situation	5.521	4.959	0.000

R^2^ = 0.173, adjusted R^2^ = 0.171, F = 26.025, *P* < 0.001.

## 4 Discussion

### 4.1 The current situation of nurses who are standardizing their training experiencing bullying in the workplace

In this study, the total score of workplace bullying was (28.10 ± 8.99), and the average score of each dimension item was: personal related negative behavior (1.20 ± 0.37), work-related negative behavior (1.32 ± 0.47), and the organizational injustice score (1.34 ± 0.49). Bullying in the workplace dimensions, the highest score is organizational injustice and the lowest is individual related bullying. In the highest score of five items, there are three from the organization injustice dimension. Scored the highest two items, respectively, “you bear overload workload,” “arrange you to do below ability level,” all from organization injustice dimension, it can be seen that nurses who are standardizing training are not valued in clinical practice and are often treated unfairly, in addition to a variety of daily tedious nursing work, may also be the teacher arranged to do not belong to their work category, below their ability level of work. In the long run, it may cause excessive pressure, even a sense of job burnout, and loss of enthusiasm and motivation for work. Low level work arrangement may not be able to fully exercise the professional ability of nurses and limit the professional ability of nurses. Nursing managers should improve the teaching plan of department training nurses, improve the selection criteria of teachers, ensure that they have excellent comprehensive quality and strong sense of responsibility, and can treat nurses who receive standardized training fairly and equally. In this way, the standardizing training nurses can be helped to improve their nursing skills and productivity, and encourage them to undertake more nursing tasks within their capacity. At the same time, we should pay attention to the physical and mental health of the nurses and create a harmonious cultural environment of the department.

### 4.2 Potential categories of workplace bullying

Current studies of workplace bullying are mostly variable-centric analysis and do not consider heterogeneity between individuals. Indeed, the types of workplace bullying experienced among nurses with equal scores may be different, and there may be group heterogeneity in nurses’ workplace bullying. This study used the potential profile analysis method, combined with various indicators, to prove the reliability of the three classification results of the potential profile analysis. The three potential categories of workplace bullying for standardized training of nurses were: group suffering severe workplace bullying (C1), group not experiencing workplace bullying (C2), and group experiencing adverse workplace situations (C3).

The average score of nurses in the “Severe workplace bullying group” (C1) in the NAQ-R dimension was 3.24, 3.78, and 3.50, both higher than 2 and higher than nurses in the “No workplace bullying group” (C2) and “Confronted with adverse workplace situation group” (C3). In this study, nurses in this category accounted for 1% of the total number of nurses, with the smallest proportion. To further reduce bullying in the workplace, hospitals should establish clear anti-bullying policies, strengthen training, and raise staff awareness. The nurses themselves should speak out bravely and face it actively, and safeguard their own reasonable rights and interests. Managers should deal with disputes in a timely manner, arrange work tasks fairly, treat nurses at each level equally, and create a harmonious, respectful and mutual aid working environment.

(C2) nurses in the group had 542 nurses, accounting for 84%, the largest proportion, and the average score of nurses in the three dimensions of workplace bullying was 1.08 points, 1.15 points and 1.19 points, respectively, all less than 2 points. Some studies have found that bullying in the workplace is hidden, as some nurses may not report (Somani et al., 2021) for concerns about affecting their career prospects or relationships in the workplace. Nursing managers should establish confidential and safe reporting channels, and make it clear that they will seriously deal with the bullies, and give support and protection to the bullied nurses. At the same time, relevant training is carried out to make the regulation training nurses realize the importance of reporting, allay a fear, and bravely protect their own rights and interests.

The average score of NAQ-R items in “Confronted with adverse workplace situation group” (C3) was 1.75, 2.03, and 2.02, among them, work-related negative behavior and organizational injustice dimension scored more than 2 points, moreover, the total score and all dimensions of workplace bullying were significantly lower than the “Severe workplace bullying group” (C1), and significantly higher than the “No workplace bullying group” (C2). The proportion of standardizing training nurses in the “adverse workplace situation group” (C3) is 15%, which is lower than the study of Ji Yuanyuan and others. The reason may be that the culture in Sichuan is relatively peaceful and inclusive, and people pay more attention to harmonious interpersonal relations. In the workplace, they may tend to solve problems through communication and negotiation, which reduces the occurrence of bullying. However, Beijing, as the national capital and an international metropolis, has a fast pace of work and high competitive pressure, which may be more likely to cause interpersonal tension and conflict to some extent. In addition, hospitals in Sichuan province may pay more attention to humanistic care and team building in nurse management. Through various training and activities, enhance the cohesion and cooperation among nurses, and adopt a zero tolerance attitude towards bullying, so that the bullying phenomenon can be better contained. However, some hospitals in Beijing may be relatively difficult to manage due to their large size and large staff, and inadequate measures to prevent bullying in the workplace. Some nurses believe that the harm caused by workplace bullying to standardized training of nurses will accumulate (Syed et al., 2022). Therefore, nursing managers should develop a scientific work allocation system, arrange tasks according to their ability and needs, and avoid overwork of standardized training of nurses. Establish feedback channels, so that the standardized training nurses can reflect the problems in time. Create a fair and inclusive team atmosphere, and seriously deal with violations, so as to ensure the physical and mental health of standardized training nurses.

### 4.3 There are differences in demographic characteristics of potential categories of workplace bullying

The results of this study indicate that demographic characteristics such as age, average weekly work hours, love of nursing, harmony with colleagues, and formal work before training influence the potential categories of workplace bullying among training nurses. The current study showed that age was significantly associated with the potential category of workplace bullying (*p* = 0.000), with young nurses aged ≤20 suffering significantly less workplace bullying than those aged 21 to 25. Probably because regulation nurses aged under 20 years are usually in the transition stage from adolescence to early adulthood, and individuals at this stage are often prone to show submissive and adaptive (Fang, 2021). They may be more inclined to follow instructions from their superiors and be relatively less sensitive to the demands and pressures of the workplace environment, thus reducing the possibility of conflict with others. Standardized nurses aged 21 to 25 are in early adulthood, more self-aware and may be more courageous to express their views and needs, which may partly increase the risk of conflicts with others. The average weekly work time was significantly associated with the potential category of workplace bullying (*p* = 0.000), and the longer the average weekly work time, the greater the proportion of severe workplace bullying groups. With the increase of working hours, the work pressure faced by the regular training nurses is constantly accumulating. The heavy work tasks, the diversification of patients’ needs, and the existence of medical risks all make the training nurses are under great psychological pressure when working for a long time. When stress exceeds a certain limit, they are less tolerant of their surroundings and are more likely to feel negative behaviors in the workplace and attribute them to workplace bullying (Chen Y. H. et al., 2022). The love of nursing was significantly associated with the potential category of workplace bullying (*p* = 0.000), Nursing-loving nurses perceived significantly less bullying in the workplace than those who were general and not passionate about nursing work. This is consistent with the study of Xu (2021). Standardized training nurses who love nursing work usually devote themselves to their work and focus on providing quality nursing services to patients. They focused more on improving their professional skills and caring for patients, but less on interpersonal conflict or bad behavior at work. In contrast, regular nurses who do not love or have a general attitude towards nursing may be more easily affected by negative factors at work and be more sensitive to possible bullying (Zhao and Li, 2020). In addition, nurses who love nursing work are often more willing to cooperate with their colleagues and actively establish good interpersonal relationships. Their enthusiasm and positive attitude may infect others, making the interaction between colleagues more harmonious. Even with some small friction, it is easier to resolve through communication and understanding, thus reducing the number of situations identified as bullying in the workplace. However, regular nurses who have a general or unloving attitude towards nursing work may be relatively negative in interpersonal relationship processing, which is more likely to cause conflicts and conflicts. Harmony with colleagues was significantly associated with the potential category of workplace bullying (*p* = 0.000), Nurses with close relationships with colleagues had significantly perceived less workplace bullying than those with average or poor relationships with colleagues. Nurses who work well with colleagues can get emotional support from colleagues in their work. When faced with situations that may be considered workplace bullying, they feel the strength to reduce the perceived intensity of these negative situations. At the same time, having a harmonious relationship means having a good communication channel. In case of problems or conflicts, the regular nurses who have a good relationship with colleagues can communicate with colleagues in time and resolve potential conflicts through mutual communication (Galanis et al., 2024). This avoids escalating minor problems to workplace bullying. When the relationship is general or not harmonious, the communication is often blocked, which easily leads to the deepening of misunderstanding and contradictions, and then increases the generation of bullying in the workplace. Whether they had done formal work before the training was significantly related to the potential category of workplace bullying (*p* = 0.008). The proportion of the nurses who had formally worked before the training was higher than those who had not formally worked before the training. The reason may be related to the fact that nurses who worked before the training may have formed certain working habits and expectations. In the regulatory environment, there may be new job requirements and interpersonal relationships, prone to adaptive difficulties, thus increasing the perception of bullying. However, the nurses who have not worked formally, like a blank sheet of paper, are easier to adapt to the new environment, have relatively low requirements and expectations for the workplace, and are less easy to perceive bullying in the workplace.

### 4.4 Current status of nurses’ spiritual climate

The spiritual climate score of 648 nurses in Sichuan province (15.24 ± 3.13), compared with the middle score (11.00), at the above-average level, was higher than the research results of (Chen X. et al., 2022), indicating that the nurses had a good atmosphere, more career development opportunities and less psychological pressure. In this study, the highest score on the spiritual climate scale was “In my department, everyone can express their own ideas, we respect each other and understand each other.” This represents that the department has an open, inclusive and harmonious atmosphere. It shows that the nurses can freely express their ideas and promote communication and collaboration. This environment helps to improve job satisfaction and team cohesion (Huang et al., 2021). The spiritual climate of the department is very important to train nurses. A good spiritual climate of the department can create a harmonious atmosphere, promote teamwork, but also reduce the psychological pressure of nurses, provide growth opportunities for nurses, enhance the professional identity of nurses, and provide better nursing services for patients.

### 4.5 Relationship between potential categories of workplace bullying and spiritual climate

The linear regression analysis of this study showed that the degree of workplace bullying in Sichuan province was negatively correlated with the spiritual climate (r = 0.171, *p* < 0.001), which indicated that the higher the perception of bullying in the workplace, the lower the degree of spiritual climate, because bullying in the workplace will bring great psychological pressure to the nurses. Nurses who are being bullied may develop negative emotions such as anxiety, fear, and depression. These emotions can affect their perception and feelings of the work environment, and reduce their psychological security and trust. When training nurses perceive being bullied, they may lose enthusiasm and motivation for their work and feel confused and helpless about their career development. This negative work attitude can further influence their assessment of the workplace spiritual climate. In addition, bullied regulation nurses may develop hostility and mistrust of their colleagues, thus reducing communication and cooperation with their colleagues. This strained interpersonal relationship can affect the cohesion and collaboration of the whole team and reduce the degree of spiritual climate in the workplace.

## 5 Conclusion

There is group heterogeneity of workplace bullying among standardized training of nurses, which can be divided into three potential categories: “Severe workplace bullying group,” “No workplace bullying group” and “Adverse workplace situation group.” The low proportion of bullying among regular training nurses in Sichuan province may be related to the unique life culture characteristics of Sichuan province. Among the nurses who suffer from workplace bullying, most of the bullying behaviors come from the organizational injustice, which reminds nursing managers and educators to pay great attention to this phenomenon, understand the causes, specific forms and negative effects of bullying in the workplace, and take effective measures to ensure that the nurses are treated fairly. Demographic characteristics such as age, average weekly work hours, love for nursing, good relationships with colleagues, and formal work before training affect the potential categories of workplace bullying. It is suggested that nursing managers should understand the relevant situation of regular training nurses, improve the work system of the department, properly assign work tasks, and reduce the occurrence of bullying in the workplace. Sichuan province standardized training nurses spiritual climate score is higher, and a negative relationship with workplace bullying, prompt nursing managers should establish a good communication mechanism, strengthen team building, strengthen the anti-bullying policy, and focus on the standardized training nurses physical and mental health, to reduce the incidence of standardized training nurses workplace bullying, improve the department spiritual climate score.

## Data Availability

The raw data supporting the conclusions of this article will be made available by the authors, without undue reservation.
